# Informed decision making before changing to RDT: a comparison of microscopy, Rapid Diagnostic Test and molecular techniques for the Diagnosis and Identification of Malaria Parasites in Kassala Eastern Sudan

**DOI:** 10.1186/1475-2875-9-S2-P32

**Published:** 2010-10-20

**Authors:** Mamoun MM Osman, Bakri YM Nour, Mohamed F Sedig, Laura de Bes, Adil M Babikir, Ahmed A Mohamedani, Petra F Mens

**Affiliations:** 1 Faculty of Medicine, Kassala University, Kassala, Sudan; 2 Faculty of Medical Laboratory Sciences, University of Gezira, Wad Medani, Sudan; 3 Blue Nile Institute for Communicable Diseases, University of Gezira, Wad Medani, Sudan; 4 Royal Tropical Institutes (KIT) Biomedical Research, Amsterdam, The Netherlands

## 

Rapid diagnostic tests are promoted for the diagnosis of malaria in many countries. The question rises whether laboratories where the current method of diagnosis is microscopy should also switch to RDT. This problem was studied in Kassala, Sudan where the issue of switching to RDT is under discussion.

Two hundreds and three blood samples were collected from febrile patients suspected of having malaria. These were subsequently analyzed with microscopy, RDT (SD Bioline P.f/P.v) and PCR for the detection and identification *of Plasmodium* parasites.

Malaria parasites were detected in 36 blood samples when examined microscopically, 54 (26.6%) samples were found positive for malaria parasites by RDT and 44 samples were positive with PCR.

Further analysis showed that the RDT used in our study resulted in a relatively high number of false positive samples. When Microscopy was compared to PCR and agreement of 96.1 % and k=0.88 (sensitivity 85.7%, specificity 100%) was found. However, when RDT was compared to PCR an agreement of only 81.2 and k=0.48 (sensitivity 69%, specificity 84%) was found. PCR has proven to be one of the most specific and sensitive diagnostic methods, particularly for malaria cases with low parasitaemia. However this technology has limitations in its routine use under resource-limited conditions, such as our study location. At present based on these results, microscopy remains the best option for routine diagnosis of malaria in Kassala, eastern Sudan.

